# Raw and *Sous-Vide*-Cooked Red Cardoon Stalks (*Cynara cardunculus* L. var. *altilis* DC): (Poly)phenol Bioaccessibility, Anti-inflammatory Activity in the Gastrointestinal Tract, and Prebiotic Activity

**DOI:** 10.1021/acs.jafc.1c03014

**Published:** 2021-08-04

**Authors:** Estíbaliz Huarte, Gessica Serra, Andrea Monteagudo-Mera, Jeremy Spencer, Concepción Cid, María-Paz de Peña

**Affiliations:** † Departamento de Ciencias de la Alimentación y Fisiología, Facultad de Farmacia y Nutrición, Universidad de Navarra, C/ Irunlarrea 1, 31008 Pamplona, Spain; ‡ Department of Food and Nutritional Sciences, University of Reading, Whiteknights, P.O. Box 226, RG6 6AP Reading, U.K.; § IdiSNA, Navarra Institute for Health Research, C/ Irunlarrea 1, 31008 Pamplona, Spain

**Keywords:** Cynara, heat treatment, gastrointestinal digestion, (poly)phenols, cytokines, gut microbiota

## Abstract

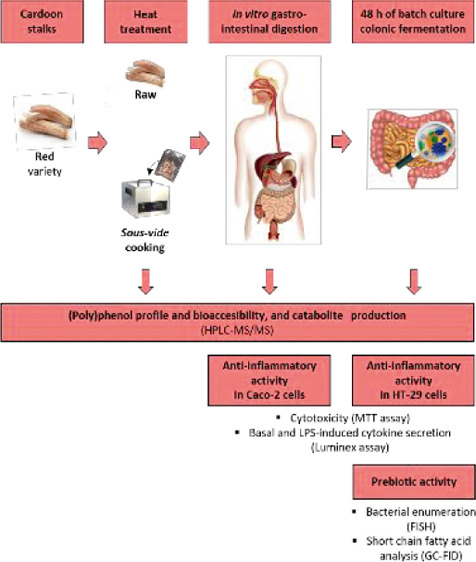

The *in vitro* anti-inflammatory and prebiotic activity and the content and profile of bioaccessible (poly)phenols and catabolites of raw and *sous-vide*-cooked red cardoon (*Cynara cardunculus* L. var. *altilis* DC) were investigated during gastrointestinal (GI) digestion. Raw cardoon after *in vitro* GI digestion had 0.7% bioaccessible (poly)phenols, which protected against lipopolysaccharide-induced inflammation by counteracting IL-8, IL-6, TNF-α, and IL-10 secretions in differentiated Caco-2 cells. Contrarily, GI-digested s*ous vide* cardoon showed higher (poly)phenol bioaccessibility (59.8%) and exerted proinflammatory effects in Caco-2 cells. (Poly)phenols were highly metabolized during the first 8 h of *in vitro* fermentation, and nine catabolites were produced during 48 h of fermentation. Colonic-fermented raw and *sous-vide*-cooked cardoon did not show anti-inflammatory activity in HT-29 cells but presented potential prebiotic activity, comparable to the commercial prebiotic FOS, by stimulating health-promoting bacteria such as *Bifidobacterium* spp. and *Lactobacillus*/*Enterococcus* spp. and by increasing the production of total SCFAs, especially acetate.

## Introduction

Diets consisting of low amounts of fruits, vegetables, and other fiber- and prebiotic-rich foods and high amounts of refined grains, alcohol, and ultra-processed foods are associated with changes in the gut microbiota composition and function (namely, dysbiosis) and lead to systemic chronic inflammation (SCI).^1^ In addition, there is evidence that SCI is implicated in disease onset or progression of metabolic syndrome.^1^ Therefore, it is of great importance to find nutritional or therapeutic interventions that help prevent or reduce an inflammatory state and maintain healthy microbiota.

Cultivated cardoon (*Cynara cardunculus* L. var. *altilis* DC), a Mediterranean plant belonging to the family Asteraceae, is mainly cultivated in Spain, Italy, Greece, France, and south Portugal. Cardoon stalks are usually consumed raw in salads or in a boiled form. Although the white variety is better known, stalks from the red cardoon variety are a richer source of (poly)phenols than those of the white variety.^2^ In addition, the application of heat treatments, such as frying and griddling, on cardoon stalks exerts a positive effect on the bioaccessibility of (poly)phenols, resulting in higher (poly)phenol bioaccessibility in digested cooked cardoons than in the raw ones after gastrointestinal (GI) digestion.^3^ Furthermore, the innovative culinary technique of *sous vide* cooking promoted a greater amount of caffeoylquinic acids (CQAs) both before and after GI digestion than traditional boiling in other *Cynara* vegetable-like globe artichoke.^4^

About 90% of dietary (poly)phenols, along with nondigestible carbohydrates and other plant components (such as lignin, resistant proteins, and carotenoids), are resistant to digestion and absorption in the small intestine.^5^ Thus, they reach the colon where they can be completely or partially hydrolyzed into smaller and more absorbable compounds by the gut microbiota. In a previous study on white cardoon stalks, gut microbiota action induced the formation of a higher total amount of the (poly)phenol-derived catabolites [protocatechuic acid, dihydrocaffeoylquinic, dihydrocaffeic, and 3-(3-hydroxyphenyl)propionic acids] in fried and griddled cardoon stalks than in raw ones during an *in vitro*-simulated colonic fermentation.^3^ Thus, different bioaccesibilities of bioactive compounds and their catabolites between raw and cooked vegetables could impact their potential biological activity in the GI tract.

There is growing evidence of the ability of (poly)phenols to modulate inflammation, the gut microbiota composition, and metabolic activity.^6−10^ Then, throughout the upper and lower GI digestion processes, red cardoon bioactive compounds, such as (poly)phenols, and/or their resulting catabolites, might exert anti-inflammatory and/or prebiotic activity on the small intestine and/or the colon, before being absorbed into the blood or excreted. To the best of the authors’ knowledge, there are no previous studies that evaluate the biological properties of red cardoon and neither the impact of culinary treatments and the GI digestion on its potential bioactivity. Therefore, this study aimed to investigate raw and *sous-vide*-cooked red cardoon stalks at different stages of *in vitro* upper and lower digestions (simulated oral-GI digestion and colonic fermentation) in terms of their (1) (poly)phenol bioaccessibility and metabolization by gut microbiota; (2) anti-inflammatory activity in the small intestine (differentiated Caco-2 cells) or colon (HT-29 cells); and (3) potential prebiotic activity on fecal human microbiota.

## Materials and Methods

### Chemicals and Reagents

Two plants of red cardoon stalks (*C. cardunculus* L. var. *altilis* DC) of approx. 50 cm in height and around 2.5 kg each, and harvested the day before, were purchased in a local retail store during the winter season (January 2019). Peptone water, yeast extract, bile salts, and phosphate buffered saline (PBS) tablets were obtained from Oxoid Ltd (Basingstoke, UK). Methanol, acetonitrile and 98% formic acid were acquired from PanReac AppliChem (Darmstadt, Germany). Pure phenolic standards were purchased from Sigma-Aldrich (Steinheim, Germany), except for dihydrocaffeic acid (Alfa Aesar, Thermo Fisher Scientific, Massachusetts, USA), the dicaffeoylquinic acids (diCQAs) 1,4-diCQA and 4,5-diCQA (MedChem Express, New Jersey, USA), and 3,5-diCQA and apigenin (Extrasynthese, Genay, France). Low glucose (1 g/L) Dulbecco’s modified Eagle’s medium (DMEM) GlutaMAX containing pyruvate with and without phenol red; high glucose (4.5 g/L) DMEM with and without phenol red; Dulbecco’s PBS; 0.25% trypsin–ethylenediaminetetraacetic acid (1×); penicillin–streptomycin; heat-inactivated fetal bovine serum (FBS); and MEM nonessential amino acids (100×) were obtained from Gibco (Paisley, UK). The standard fructooligosaccharide (FOS) OraftiP95 was purchased from BENEO-Orafti (Oreye, Belgium). Digestive enzymes, lipopolysaccharide (LPS) from *Escherichia coli* 026:B6, 3-(4,5-dimethylthiazol-2-yl)-2,5-diphenyltetrazolium bromide (MTT), oligonucleotide probes, and other chemicals and reagents used were purchased from Sigma Aldrich (Poole, UK).

### Red Cardoon Sample Preparation

Cardoon stalks were washed, and the spiny skin was removed manually. Then, they were cut into rectangular homogeneous pieces (1.5 × 6 cm approx.), manually mixed together, and divided into two parts (1.8 kg each). One-half was named raw cardoon and lyophilized in a freeze dryer Cryodos-80 (Telstar, Terrasa, Spain). The other half was packaged into a total of 33 vacuum bags, each one containing around 55 g of fresh cardoon stalks and a thin layer of water (45 mL). The bags were then vacuum-sealed by using a vacuum sealer (VP-3710.10 AK-Ramon, Vilassar de Dalt, Barcelona, Spain), immersed in boiling water (98 °C), and then maintained at 85 °C for 50 min. Water temperature was controlled with a temperature probe. All *sous-vide*-cooked cardoon stalks were lyophilized in a freeze dryer Cryodos-80 (Telstar, Terrasa, Spain), then crushed with a chopper (Moulinex, Barcelona, Spain), and pooled to have a representative sample of *sous-vide*-cooked cardoon, avoiding thermal process variability. Lyophilized raw and *sous-vide*-cooked cardoon samples were stored at −18° C until analysis.

### Simulated Oral-GI Digestion

Red cardoon samples underwent an oral-GI *in vitro* digestion according to the method described by Mills et al.^11^ with some modifications. Briefly, 350 mL of distilled water were added to 35 g of each freeze-dried sample in a glass screw topped bottle placed in a room at 37 °C. The bottle was shaken and connected to a pH sensor. Three sequential steps were performed in the absence of light. First, the oral digestion was performed by adding 3.65 mL of the α-amylase solution from *Bacillus licheniformis* (47.9 U/mL) to each bottle and then, the sample was shaken for 30 min. Second, the gastric digestion step was carried out by adding 14.6 mL of a porcine pepsin solution (589.2 U/mL), and distilled water up to 650 mL. Samples were shaken for 2 h maintaining pH 3 by adding 6 M HCl. Finally, the intestinal step was accomplished by adding 72.9 mL of a porcine pancreatin solution (33 U trypsin/mL) with bile extracts (2.76 mg/mL). Samples were shaken for 2 h at pH 7 by the addition of 6 M NaOH. The enzymatic digestive extract of each cardoon sample was divided into 3 fractions. The first fraction (300 mL) was freeze-dried (ScanVac CoolSafe, Labogene, Lillerød, Denmark) and stored at −80 °C until (poly)phenol analysis, and the second fraction (150 mL) was used for analysis of cytotoxicity and impact on cytokine secretion in differentiated Caco-2 cells. The third fraction (220 mL) was transferred into a cellulose dialysis membrane (100–500 Da molecular weight cutoff, 1.8 mL/cm, 24 mm, 100 M Spectra/Por Liotech) (Fisher Scientific, Loughborough, UK) and dialyzed overnight at 4 °C against 0.01 M NaCl (4 L) to remove low-molecular-weight digestion products and monosaccharides. The dialysis fluid was changed and dialysis continued for an additional 2 h. The digest retained in the dialysis tube represent the nonabsorbed sample in the small intestine that might pass to the colon and be fermented by gut microbiota. This retained digest was freeze-dried (ScanVac CoolSafe, Labogene, Lillerød, Denmark) and stored at −18 °C until fermentation, or at −80 °C until (poly)phenol analysis.

### Fecal Sample Preparation

Human fecal samples from three healthy volunteers were collected on the day of the inoculation of the batch culture vessels in anaerobic jars (Anaerojar TM 2.5L, Oxoid Ltd), which included a gas-generating kit (AnaeroGen TM, Oxoid). They were processed within 1 h of bowel movement. All the volunteers had not taken any antibiotics for at least 6 months before the study and had no history of bowel or GI disease and followed a polyphenol-free diet (avoiding fruits and vegetables, nuts, legumes, high-fiber products, and beverages such as tea, coffee, and fruit juices, as well as alcohol) for 2 days before fecal collection. Fecal slurry from each individual was prepared by homogenizing human feces (10% w/v) in PBS (0.1 M; pH 7.4) in a stomacher (Stomacher 400, Seward, Norfolk, UK) at 460 paddle beats/min for 2 min.

### Simulated Colonic Fermentation

GI-digested and dialyzed cardoon samples were submitted to an *in vitro*-simulated colonic fermentation using fecal batch cultures. In order to mimic conditions located in the distal region of the human large intestine, the experiment was run under anaerobic conditions at 37 °C and pH 6.7–6.9 for a period of 48 h. Briefly, sterilized glass water-jacketed vessels (300 mL) were aseptically filled with a presterilized basal nutrient medium (135 mL) containing the following: peptone water (2 g/L), yeast extract (2 g/L), NaCl (0.1 g/L), K_2_HPO_4_ (0.04 g/L), KH_2_PO_4_ (0.04 g/L), MgSO_4_·7H_2_O (0.01 g/L), CaCl_2_·6H_2_O (0.01 g/L), NaHCO_3_ (2 g/L), Tween 80 (2 mL/L), hemin (0.05 g/L), vitamin K1 (10 mL/L), l-cysteine (0.5 g/L), bile salts (0.5 g/L), resazurin (1 mg/L), and distilled water. The vessels were then gassed overnight with O_2_-free N_2_ (15 mL/min) and magnetically stirred. The following day, the temperature of the vessels was set to 37 °C by use of a circulating water bath, and the pH was maintained at 6.7–6.9 using an Electrolab pH controller (Tewksbury, UK). Then, 1.5 g of each freeze-dried digested sample (1% w/v final volume) was added to the stirred vessels just before the addition of 15 mL of the fecal slurry (1% w/v final volume). The prebiotic FOS OraftiP95 at 1% w/v final volume was included as the positive fermentation control, and basal nutrient media with no substrate was added as the negative fermentation control (NFC). Batch cultures were run for 48 h and were performed in triplicate with three different fecal volunteers for each substrate. Aliquots from cardoon sample and control fermentation were collected at various time points for (poly)phenol and catabolite analysis (15 mL at 0, 4, 8, 24, and 48 h), cytotoxicity and impact on cytokine secretion analysis in HT-29 cells (5 mL at 8 and 24 h), and bacterial enumeration and lactic acid and short-chain fatty acid (SCFA) analysis (5 mL at 0, 8, 24 and 48 h). Collected aliquots for the analysis of (poly)phenols and catabolites were immediately inactivated with 50 μL of 33% HCl, then freeze-dried (Cryodos-80, Telstar, Terrasa, Spain), and stored at −80 °C.

### Analysis of (Poly)phenols and Catabolites by High-Performance Liquid Chromatography with Tandem Mass Spectrometry

(Poly)phenols and catabolites were extracted from the undigested, digested (with and without subsequent dialysis), and fermented cardoon samples, following the Sánchez-Salcedo et al. procedure^12^ with some modifications. Briefly, 25 mg of each freeze-dried sample were extracted with 0.5 mL of 80:20 (v/v) methanol/acidified water (0.1% formic acid) and sonicated for 90 min. Afterward, the mixture was centrifuged for 10 min at 19,956*g*. The supernatant was collected and the residue was re-extracted using 0.25 mL of 80:20 v/v methanol/acidified water, sonicated for 25 min in a sonic bath, and centrifuged for 10 min at 19,956*g*. Both supernatants were mixed, filtered with a 0.22 μm PVDF syringe filter, and stored at −18 °C until high-performance liquid chromatography with tandem mass spectrometry (HPLC–MS/MS) analysis.

(Poly)phenols and catabolites in the extracts were analyzed using an HPLC unit model 1200 (Agilent Technologies, Palo Alto, CA, USA) equipped with a triple quadrupole linear ion-trap mass spectrometer (3200 Q-TRAP, AB SCIEX). The column used was a CORTECS C18 (3 × 75 mm, 2.7 μm) from Waters. The HPLC separation was carried out using 0.1% v/v formic acid in water (solvent A) and acetonitrile (solvent B) at a constant flow rate of 0.6 mL/min and a column oven temperature of 20 °C. The injection volume was 5 μL. The mobile phase program was established as follows: 0–1 min, 5% B; 1–5 min, 5–10% B; 5–8 min, 10–20% B; 8–10.5 min, 20% B; 10.5–16 min, 20–30% B; 16–17.6 min, 30–100% B; 17.6–25.6 min, 100% B, then returned to 5% B in 4.8 min and maintained an isocratic elution until the end of the analysis (35 min). For the identification and quantification of (poly)phenols and catabolites, the ion multiple reaction monitoring mode was used. The MS worked in the negative ionization mode, with a source temperature of 600 °C and the IonSpray voltage of −3500 V. Nitrogen was used as nebulizing, turbo heater, and curtain gas and it was set at the pressures of −60, −65, and −35 psi, respectively. The declustering and entrance potentials were set at −20 and −10 V, respectively. The collision energy was optimized for each compound using the same standards as those used for identification. Compounds were identified by comparing the MS/MS fragmentation pattern and retention time with pure reference standards when available. When no standard was available, compounds were tentatively identified by comparing the MS/MS fragmentation with the literature fragmentation pathway. The retention time and mass spectrometric characteristics of (poly)phenols and catabolites identified as well as the pure reference standards used are shown in Table S1.

(Poly)phenols and catabolites were quantified by using the calibration curves of their own pure reference standard when available. For those compounds without a pure reference standard, 1-CQA and CQA derivatives I–III were quantified as 5-CQA equivalents; diCQA glucosides I–II and succinyldiCQAs I–II as 4,5-diCQA equivalents; caffeoyl-hexoside was quantified as isoquercitrin equivalents; and luteolin acetylglucoside was quantified as luteolin 7-*O*-glucoside equivalents. The limit of quantification (LOQ) was 0.25 μg/mL for hesperidin and phenylacetic acid; 0.05 μg/mL for 1,3-diCQA, isoferulic acid, quercetin, 3-hydroxyphenylacetic acid, protocatechuic acid, and 4-hydroxybenzoic acid; and 0.025 μg/mL for the rest of the standards used. Chromatograms and spectral data were acquired using Analyst software 1.6.3 (AB SCIEX). Those polyphenols or catabolites detected and quantified in the NFC samples were subtracted from the colonic fermented cardoon samples. Each sample was analyzed in duplicate. The results are presented as the mean of micrograms of (poly)phenol per gram of the cardoon sample dry matter (μg/g dm) ± standard deviation (SD).

The bioaccessibility of (poly)phenols from digested (with and without subsequent dialysis) and fermented cardoon samples was determined as follows:
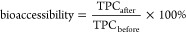
where TPC_before_ is the total poly(phenol) content before digestion and TPC_after_ is the total poly(phenol) content after digestion/dialysis/fermentation.

### Cell Culture Conditions and Treatments

Caco-2 cells (human colon adenocarcinoma cells) were purchased from the European Collection of Cell Cultures (ECACC) (Salisbury, Wiltshire UK) and cultured in low glucose (1 g/L) DMEM containing pyruvate and glutamine and supplemented with 1% (v/v) of nonessential amino acids. HT-29 cells (human colon adenocarcinoma cell line) were obtained from ECACC and cultured in high glucose (4.5 g/L) DMEM containing l-glutamine. Cell media from both cell lines were supplemented with 10% v/v FBS, 100 U/mL penicillin, and 0.1 mg/mL streptomycin. Cells were incubated in a humidified atmosphere at 5% CO_2_ and 37 °C and sub-cultured 2–3 times per week.

For experiments with Caco-2 cells, fractions collected after the digestion of cardoon samples were immediately centrifuged at 1543.5*g* for 15 min, and the resulting supernatants were collected and 0.45 μM filtered to separate the soluble fraction that might be available for absorption into intestinal cells (bio-accessible fraction). For experiments with HT-29 cells, aliquots collected during the fermentation of cardoon samples or from the NFC of the three volunteers at the same fermentation time (8 or 24 h) were combined and centrifuged (1543.5*g*, 10 min). Then, supernatants were 0.45 and 0.22 μM filtered to obtain the potentially bio-accessible fraction for colon cells. The filtered supernatants of the digestion and fermentation of cardoon samples and the NFC were divided in small aliquots (1.5 mL) and stored at −20 °C for use.

Cells were seeded in 96-well plates (3.4 × 10^4^ cells/mL for Caco-2 cells and 2.2 × 10^5^ cells/mL for HT-29 cells; 150 μL/well) for the MTT assay and in 24-well plates (3.12 × 10^4^ cells/mL for Caco-2 cells and 1.84 × 10^5^ cells/mL for HT-29 cells; 1 mL/well) for the Luminex assay. Caco-2 cells were allowed to grow to confluent monolayers for 19 days at 37 °C and 5% CO_2_ prior to the experiments in order to differentiate and develop an enterocyte-like phenotype. Their cell media were changed every 3 days. HT-29 cells were incubated for 24 h at 37 °C and 5% CO_2_, reaching a sub-confluent state prior to the experiments.

Different LPS concentrations and exposure times (0.1–50 μg/mL, 24–72 h) were preliminarily analyzed in both cell lines, and in undifferentiated Caco-2 cells, for their cytotoxicity (MTT assay) and impact on cytokine secretion (Luminex multiplex assay). Undifferentiated Caco-2 cells were dismissed due to their undetectable [interleukin (IL)-1β, IL-6, IL-10, and tumor necrosis factor (TNF)-α] or very low (IL-8) capacity to secrete cytokines in response to LPS. LPS at 5 and 0.1 μg/mL in the differentiated Caco-2 and HT-29 cells, respectively, and 48 h of exposure were selected as the treatment to induce an inflammatory state in cells on the basis of its ability to stimulate high cytokine secretion without being cytotoxic. Furthermore, the cytotoxicity of supernatants of cardoon sample digestion (2.5–20% v/v in cell media, which corresponds to 1.21–9.68 mg dm/mL) or fermentation (0.1–10% v/v in cell media, which corresponds to 9.53–953.2 μg dm/mL) was previously analyzed by the MTT assay in both cell lines. Then, noncytotoxic concentrations were selected for the assessment of the anti-inflammatory potential, which were 2.42 and 9.68 mg cardoon dm/mL for the digested cardoon samples (≥88% MTT reduction relative to the control, both in the absence and in the presence of 5 μg/mL LPS) (Figure S1a) and 23.8 and 95.3 μg cardoon dm/mL for fermented cardoon samples (>80% MTT reduction relative to the control, both in the absence and in the presence of 5 μg/mL LPS) (Figure S1b).

Once the concentrations and exposure times were optimized, the experiments were carried out under the following conditions: on the day of the experiment, the cell medium was removed, and differentiated Caco-2 cells were washed with phenol red-free DMEM at 2.5% FBS and HT-29 cells with PBS. Differentiated Caco-2 cells were pretreated for 1 h with two selected supernatant concentrations of the GI-digested cardoon samples (2.42 and 9.68 mg cardoon dm/mL which are equivalent to 5 and 20% v/v in cell media, respectively). Besides, HT-29 cells were pretreated for 1 h with two selected supernatant concentrations of 8 and 24 h colonic-fermented cardoon samples (23.8 and 95.3 μg cardoon dm/mL, which are equivalent to 0.25 and 1% v/v in cell media, respectively) and two supernatant concentrations of 8 and 24 h NFC (0.25 and 1% v/v in cell media). Treatments continued in both cell lines for a further 48 h in the absence and presence of LPS at 5 and 0.1 μg/mL for differentiated Caco-2 and HT-29 cells, respectively. Blank controls were introduced in the experiments with both differentiated Caco-2 cells and HT-29 cells consisting of cells incubated with phenol red-free cell media at 2.5% FBS.

### Cytokine Quantification by the Luminex Multiplex Assay

After the aforementioned treatments (see section “Cell Culture Conditions and Treatment”), well contents were removed, transferred to Eppendorf tubes, and stored at −20 °C. The day of the analysis, tubes were defrosted and centrifuged at 16,000*g* for 5 min (Centrifuge Thermo Scientific, Heraeus Fresco 17, Massachusetts, United States). The concentration of IL-8, IL-6, IL-1β, TNF-α, and IL-10 on the diluted supernatants was measured using a bead-based Luminex multiplex assay (Human High Sensitivity Cytokine Premix Kit A, Magnetic Luminex High Performance Assay, R&D Systems, United Kingdom) following the manufacturer’s instructions. The analysis was carried out with a Luminex 200 System and Luminex Xponent software (Luminex Corporation, Austin, Texas, USA). Three independent experiments were performed.

### Enumeration of Bacterial Populations by Fluorescence *In Situ* Hybridization

Aliquots (750 μL) collected from the fermentation of the cardoon samples were immediately centrifuged for 5 min at 11,338*g*. The pellet was resuspended in 375 μL of PBS and fixed in 1125 μL of the 4% w/v paraformaldehyde solution at 4 °C for 4 h. Fixed samples were centrifuged at 11,338*g* for 5 min, and the cell pellet was washed twice in 1 mL of sterile cold PBS (0.1 M, pH 7.0) and centrifuged at 11,338*g* for 5 min. Finally, the cell pellet was resuspended in PBS (300 μL) and 99% ethanol (300 μL) and stored at −20 °C until analysis. Then, bacterial populations were enumerated by fluorescence *in situ* hybridization (FISH) using oligonucleotide probes targeting specific regions of 16S rRNA. Probes were commercially synthesized and labeled with the fluorescent dye Cy3. The following probes were used: Bif164 for *Bifidobacterium* spp.;^13^ Lab158 for *Lactobacillus*/*Enterococcus*;^14^ Erec482 for *Eubacterium rectale*–*Clostridium coccoides* group (*Clostridium* cluster XIVa);^15^ Bac303 for *Bacteroides*–*Prevotella* group;^16^ and EUB 338 mixture consisting of EUB338, EUB338II, and EUB338III for total bacteria.^17^ Fixed samples were hybridized as described by Daims, Stoecker, and Wagner.^18^ Bacterial cells were counted in 15 random fields of view per sample using a fluorescence microscope (Nikon E400 Eclipse, Tokyo, Japan). Three independent fermentation aliquots of cardoon samples with fecal samples from 3 different donors were analyzed.

### Analysis of Lactic Acid and SCFAs by Gas Chromatography with Flame Ionization Detection

Aliquots (1 mL) collected from the fermentation of cardoon samples were immediately centrifuged (11,338*g* for 10 min) to remove bacteria and other solids. Supernatants were then transferred into clean tubes and frozen at −20 °C. Then, lactic acid and SCFAs from the fermented samples were extracted and derivatized as previously described.^19^ Briefly, 500 μL of each sample or standard solution were added to a 100 mm × 16 mm flat-bottomed glass tube with 25 μL of 0.1 M 2-ethylbutyric acid as the internal standard. Then, 250 μL of the concentrated HCl and 1.5 mL of diethyl ether were added to each tube, vortexed for 1 min, and centrifuged (10 min at 752*g*). Tubes were left at room temperature overnight and the day after, 500 μL of diethyl ether (the upper layer) were transferred into GC screw-cap vials. Then, 25 μL of *N*-(*tert*-butyldimethylsilyl)-*N*-methyltrifluoroacetamide was added to each vial with the ether extract. Then, the vials were left at room temperature for 72 h to allow lactic acid in the samples to completely derivatize. Then, a 7890B Gas Chromatograph (Agilent Technologies, Cheshire, UK) equipped with FID was used for the analysis of lactic acid and SCFAs in the fermented samples. The column used was a HP-5MS (30 m × 0.25 mm × 0.25 μm; HP-5 5% diphenyl/95% dimethylpolysiloxane) from Agilent Technologies. Both the injector and detector were held at 275 °C. The sample injection volume was 1 μL, and a split ratio of 100:1 was used. The carrier gas helium was set at 5 psi and a flow rate of 6.5 mL/min. The GC oven was held at 63 °C for 3 min, programed to 190 °C at 10 °C/min, and then held constant at 190 °C for 1 min. Lactic acid and SCFAs (acetic, propionic, butyric, isobutyric, and isovaleric acids) were quantified by using a calibration curve performed with a standard solution at concentrations of 6.25–50 mM. Peak areas were integrated and calculated using ChemStation B.01.04.232 software (Agilent Technologies, Cheshire, UK). Three independent fermentation aliquots of cardoon samples with fecal samples from three different donors were analyzed.

### Statistical Analysis

One-way analysis of variance (ANOVA) with Tukey’s *post hoc* test was conducted to determine the differences in cytokine concentrations between each treatment and control (untreated cells and NFC-treated cells; with or without LPS stimulation), as well as differences among the raw and cooked cardoon treatments at the same concentration. ANOVA and Tukey’s *post hoc* test was also applied to determine the significant differences in bacterial counts, and in lactic acid and SCFA concentrations, among treatments (NFC, FOS, raw, and *sous-vide*-cooked cardoon) at the same fermentation time points (0, 8, 24, and 48 h). Differences in bacterial counts, and in lactic acid and SCFA concentrations, from 0 h of fermentation value within the same treatment were tested using the paired Student’s *t* test. Differences were considered significant if *p* value < 0.05. All the statistical analyses were performed using the STATA v.12.0 software package.

## Results

### (Poly)phenol and Catabolite Bioaccessibility during GI-Digestion and Colonic Fermentation

A total of 19 and 30 (poly)phenols were identified and quantified in raw and *sous-vide*-cooked red cardoon (nondigested), respectively (Tables 1 and 2). Phenolic acids, and specifically hydroxycinnamic acids, represented >98% of the total content in both samples, with mono- and di-CQAs as the major (poly)phenolic compounds (43.4–48.7 and 55.5–49.2% of total polyphenols, respectively). The remaining (poly)phenols were flavonoids (apigenin, luteolin, quercetin, and hesperetin derivatives), being luteolin derivatives (luteolin 7-*O*-glucoside, luteolin 7-*O*-glucuronide, and luteolin acetylglucoside) the most abundant ones.

**Table 1:** (Poly)phenolic Profile and Catabolites from Raw and *Sous-Vide*-Cooked Red Cardoon before and after Simulated Oral GI Digestion *In Vitro*, after Subsequent Dialysis, and during a Simulated Colonic Fermentation *In Vitro* (0, 8, 24, and 48 h), as Analyzed by HPLC–MS/MS^a^

	red cardoon							
(poly)phenol	nondigested	digested	digested and dialyzed	0 h fermented	4 h fermented	8 h fermented	24 h fermented	48 h fermented
**Phenolic Acids**								
*Monocaffeoylquinic Acids (CQAs) and Derivatives*								
1-CQA								
raw	91.43 ± 5.91	2.19 ± 0.28	2.72 ± 0.11	2.16 ± 0.07	2.79 ± 0.06	3.73 ± 0.32	<LOQ	<LOQ
*sous vide*	146.99 ± 20.03	159.98 ± 33.48	218.42 ± 1.05	128.61 ± 0.92	125.39 ± 2.85	1.70 ± 0.06	0.94 ± 0.01	0.52 ± 0.08
3-CQA								
raw	105.75 ± 2.68	5.52 ± 0.91	4.14 ± 0.15	2.39 ± 0.06	2.81 ± 0.06	3.85 ± 0.41	<LOQ	nd
*sous vide*	1018.54 ± 108.87	871.63 ± 52.04	607.60 ± 11.68	356.66 ± 39.10	384.91 ± 23.89	22.01 ± 0.26	1.92 ± 0.04	<LOQ
4-CQA								
raw	14.75 ± 0.78	5.12 ± 0.56	3.07 ± 0.03	2.27 ± 0.18	2.39 ± 0.01	2.80 ± 0.26	<LOQ	nd
*sous vide*	1379.72 ± 68.27	1021.63 ± 110.42	548.55 ± 4.84	304.21 ± 6.12	273.17 ± 0.73	6.77 ± 0.02	1.83 ± 0.07	<LOQ
5-CQA								
raw	5495.39 ± 284.01	42.44 ± 1.62	31.12 ± 1.96	22.27 ± 0.13	7.61 ± 0.02	6.35 ± 0.68	0.78 ± 0.04	0.19 ± 0.04
*sous vide*	3845.58 ± 253.15	2337.01 ± 88.40	1939.52 ± 10.52	1244.25 ± 2.05	775.81 ± 8.45	7.08 ± 0.18	2.13 ± 0.01	0.31 ± 0.01
CQA Derivative I								
raw	<LOQ	nd	nd	nd	nd	nd	nd	nd
*sous vide*	6.93 ± 1.10	<LOQ	nd	nd	nd	nd	nd	nd
CQA Derivative II								
raw	<LOQ	nd	nd	nd	nd	nd	nd	nd
*sous vide*	0.71 ± 0.08	0.25 ± 0.01	0.62 ± 0.05	<LOQ	<LOQ	nd	nd	nd
CQA Derivative III								
raw	<LOQ	nd	nd	nd	nd	nd	nd	nd
*sous vide*	3.97 ± 0.57	3.36 ± 0.22	3.95 ± 0.23	1.36 ± 0.29	1.34 ± 0.12	<LOQ	nd	nd
*diCQAs and Derivatives*								
1,3-diCQA								
raw	34.75 ± 0.39	0.35 ± 0.89	1.32 ± 0.02	<LOQ	<LOQ	<LOQ	<LOQ	<LOQ
*sous vide*	2386.53 ± 210.42	1520.01 ± 105.62	687.96 ± 14.26	425.18 ± 14.90	406.11 ± 14.85	243.78 ± 3.12	6.43 ± 0.08	2.33 ± 0.29
1,5-diCQA								
raw	5151.20 ± 479.34	7.09 ± 1.43	2.63 ± 0.48	1.01 ± 0.03	1.11 ± 0.22	1.01 ± 0.00	<LOQ	nd
*sous vide*	2724.58 ± 137.79	1119.44 ± 107.59	513.46 ± 20.06	196.95 ± 65.83	175.88 ± 18.81	2.28 ± 0.15	<LOQ	nd
3,5-diCQA								
raw	1696.62 ± 109.72	3.75 ± 0.51	2.69 ± 0.08	1.58 ± 0.02	<LOQ	<LOQ	nd	nd
*sous vide*	898.67 ± 13.87	406.43 ± 23.66	255.19 ± 2.69	84.90 ± 19.35	76.29 ± 11.17	1.73 ± 0.02	<LOQ	nd
3,4-diCQA								
raw	nd	nd	nd	nd	nd	nd	nd	nd
*sous vide*	174.22 ± 4.22	118.67 ± 0.25	39.66 ± 1.34	11.44 ± 1.74	14.72 ± 0.03	1.92 ± 0.07	nd	nd
4,5-diCQA								
raw	136.51 ± 8.56	1.91 ± 0.20	1.37 ± 0.16	0.92 ± 0.01	<LOQ	nd	nd	nd
*sous vide*	217.17 ± 14.93	108.76 ± 4.33	85.06 ± 2.93	68.85 ± 1.04	80.06 ± 3.94	1.67 ± 0.04	<LOQ	<LOQ
diCQA Glucoside I								
raw	<LOQ	nd	nd	Nd	nd	nd	nd	nd
*sous vide*	1.40 ± 0.05	1.36 ± 0.00	1.40 ± 0.01	Nd	nd	nd	nd	nd
diCQA Glucoside II								
raw	1.55 ± 0.03	nd	nd	Nd	nd	nd	nd	nd
*sous vide*	1.34 ± 0.05	1.34 ± 0.00	1.40 ± 0.00	Nd	nd	nd	nd	nd
Succinyl diCQA I								
raw	139.76 ± 3.42	<LOQ	<LOQ	nd	nd	nd	nd	nd
*sous vide*	49.33 ± 5.77	16.28 ± 1.53	14.90 ± 0.15	5.72 ± 0.75	6.01 ± 0.42	<LOQ	nd	nd
SuccinyldiCQA II								
raw	147.99 ± 2.74	<LOQ	nd	nd	nd	nd	nd	nd
*sous vide*	10.83 ± 1.45	3.41 ± 0.15	3.47 ± 0.04	1.59 ± 0.12	1.66 ± 0.10	nd	nd	nd
*Other Hydroxycinnamic Acids*								
Caffeic Acid								
raw	5.43 ± 0.09	0.16 ± 0.08	0.42 ± 0.01	0.49 ± 0.09	2.92 ± 0.01	<LOQ	<LOQ	<LOQ
*sous vide*	66.72 ± 11.59	6.55 ± 0.12	0.84 ± 0.05	7.39 ± 1.34	49.27 ± 4.81	1.30 ± 0.00	2.76 ± 0.08	2.02 ± 0.35
Caffeoyl-Hexoside								
raw	1.97 ± 0.30	<LOQ	nd	nd	nd	nd	nd	nd
*sous vide*	2.49 ± 1.93	1.64 ± 1.32	0.77 ± 0.11	<LOQ	<LOQ	nd	nd	nd
Ferulic Acid								
raw	nd	<LOQ	nd	nd	nd	nd	nd	nd
*sous vide*	1.05 ± 0.07	<LOQ	nd	nd	nd	nd	nd	nd
Isoferulic Acid								
raw	nd	nd	nd	nd	nd	nd	nd	nd
*sous vide*	1.83 ± 0.57	nd	nd	nd	nd	nd	nd	nd
*p*-Coumaric Acid								
raw	<LOQ	0.85 ± 0.06	0.74 ± 0.01	<LOQ	<LOQ	<LOQ	<LOQ	<LOQ
*sous vide*	<LOQ	<LOQ	<LOQ	<LOQ	<LOQ	<LOQ	<LOQ	<LOQ
**Flavonoids**								
*Apigenin Derivatives*								
Apigenin								
raw	<LOQ	<LOQ	<LOQ	<LOQ	<LOQ	0.98 ± 0.01	nd	nd
*sous vide*	0.95 ± 0.06	0.88 ± 0.02	<LOQ	<LOQ	<LOQ	1.22 ± 0.01	nd	nd
Apigenin 7-*O*-Glucoside								
raw	0.85 ± 0.04	1.20 ± 0.02	0.14 ± 0.08	<LOQ	nd	nd	nd	nd
*sous vide*	4.34 ± 1.35	4.23 ± 0.38	1.55 ± 0.05	0.55 ± 0.03	<LOQ	nd	nd	nd
Apigenin 7-*O*-Glucuronide								
raw	2.71 ± 0.24	1.48 ± 0.07	0.60 ± 0.00	<LOQ	<LOQ	<LOQ	<LOQ	<LOQ
*sous vide*	1.16 ± 0.18	0.56 ± 0.19	<LOQ	<LOQ	<LOQ	<LOQ	<LOQ	<LOQ
Apigenin 6,8-Di-C-Glucoside (Vicenin-2)								
raw	nd	nd	nd	nd	nd	nd	nd	nd
*sous vide*	14.14 ± 1.15	8.47 ± 0.64	4.31 ± 0.01	3.66 ± 0.62	4.14 ± 0.56	2.44 ± 0.12	nd	nd
*Luteolin Derivatives*								
Luteolin								
raw	<LOQ	1.86 ± 0.05	1.41 ± 0.02	1.09 ± 0.04	2.40 ± 0.07	3.88 ± 0.18	<LOQ	<LOQ
*sous vide*	2.60 ± 0.35	1.86 ± 0.06	1.01 ± 0.12	2.62 ± 0.32	11.51 ± 0.30	21.44 ± 0.22	2.98 ± 0.14	1.32 ± 0.09
Luteolin 7-*O*-Glucoside								
raw	35.62 ± 1.21	8.73 ± 0.71	3.82 ± 0.30	3.93 ± 0.00	2.92 ± 0.18	0.37 ± 0.03	0.22 ± 0.01	0.18 ± 0.04
*sous vide*	105.01 ± 7.30	98.68 ± 5.13	48.06 ± 2.81	41.72 ± 2.68	17.04 ± 0.97	4.60 ± 0.04	4.43 ± 0.00	4.32 ± 0.05
Luteolin 7-*O*-Glucuronide								
raw	27.75 ± 0.32	2.82 ± 0.16	1.55 ± 0.03	0.81 ± 0.10	<LOQ	nd	nd	nd
*sous vide*	9.00 ± 1.09	3.51 ± 0.05	2.23 ± 0.04	0.59 ± 0.14	nd	nd	nd	nd
Luteolin Acetylglucoside								
raw	56.44 ± 2.30	7.37 ± 0.79	2.99 ± 0.18	1.40 ± 0.04	<LOQ	nd	nd	nd
*sous vide*	41.97 ± 6.06	31.56 ± 0.95	17.78 ± 0.18	5.15 ± 1.01	<LOQ	<LOQ	nd	nd
*Quercetin Derivatives*								
Quercetin								
raw	nd	nd	nd	nd	nd	<LOQ	nd	nd
*sous vide*	nd	nd	nd	<LOQ	<LOQ	1.65 ± 0.01	<LOQ	nd
Quercetin 3-Glucoside (Isoquercitrin)								
raw	2.72 ± 0.09	<LOQ	nd	nd	nd	nd	nd	nd
*sous vide*	6.63 ± 1.18	3.14 ± 0.05	1.42 ± 0.07	<LOQ	<LOQ	nd	nd	nd
*Hesperetin Derivatives*								
Hesperetin 7-Rutinoside (Hesperidin)								
raw	<LOQ	<LOQ	nd	nd	nd	nd	nd	nd
*sous vide*	11.89 ± 0.83	8.35 ± 0.14	7.91 ± 0.17	<LOQ	<LOQ	<LOQ	nd	nd
**Phenolic Catabolites**								
3-Hydroxyphenylacetic Acid								
raw	nd	nd	nd	66.61 ± 2.02	67.89 ± 1.21	83.04 ± 5.14	125.87 ± 11.14	200.29 ± 2.23
*sous vide*	nd	nd	nd	67.93 ± 3.75	75.67 ± 2.62	254.45 ± 3.25	1313.59 ± 63.63	4145.25 ± 51.05
2,5-Dihydroxybenzoic Acid								
raw	nd	nd	nd	0.45 ± 0.02	1.44 ± 0.02	2.77 ± 0.18	2.06 ± 0.03	1.64 ± 0.08
*sous vide*	nd	nd	nd	1.53 ± 0.31	2.29 ± 0.12	6.29 ± 0.08	4.14 ± 0.02	6.98 ± 0.89
3,4-Dihydroxybenzoic Acid (Protocatechuic Acid)								
raw	nd	nd	nd	nd	2.82 ± 0.13	1.50 ± 0.13	3.81 ± 0.15	6.41 ± 0.49
*sous vide*	nd	nd	nd	1.85 ± 0.41	3.89 ± 0.16	2.49 ± 0.06	1.08 ± 0.14	3.61 ± 0.93
3-(3-Hydroxyphenyl)propionic Acid								
raw	nd	nd	nd	nd	nd	4.64 ± 0.55	19.06 ± 0.55	49.54 ± 4.22
*sous vide*	nd	nd	nd	0.76 ± 1.66	4.57 ± 0.11	90.22 ± 0.89	593.52 ± 36.98	1656.57 ± 0.00
Dihydrocaffeic Acid								
raw	nd	nd	nd	nd	1.79 ± 0.08	5.04 ± 0.18	2.56 ± 0.04	1.37 ± 0.03
*sous vide*	nd	nd	nd	nd	37.31 ± 9.14	227.39 ± 4.59	2.75 ± 0.01	4.89 ± 0.45
4-Hydroxybenzoic Acid								
raw	nd	nd	nd	nd	nd	3.86 ± 0.43	5.70 ± 0.17	4.68 ± 0.43
*sous vide*	nd	nd	nd	nd	nd	3.83 ± 0.00	1.71 ± 0.31	7.35 ± 2.07
Phenylacetic Acid								
raw	nd	nd	nd	nd	nd	nd	77.14 ± 3.74	596.09 ± 14.65
*sous vide*	nd	nd	nd	nd	nd	nd	Nd	71.30 ± 24.27
1,2-Dihydroxybenzene								
raw	nd	nd	nd	nd	nd	nd	2.03 ± 0.06	2.90 ± 0.15
*sous vide*	nd	nd	nd	nd	nd	nd	1.38 ± 0.01	2.58 ± 0.43
3-Hydroxybenzoic Acid								
raw	nd	nd	nd	nd	nd	nd	nd	0.23 ± 0.07
*sous vide*	nd	nd	nd	nd	nd	nd	nd	0.46 ± 0.08

a Results are expressed as mean μg (poly)phenolic compound per g red cardoon sample dry matter ± SD. nd, not detected; <LOQ, below the limit of quantification.

**Table 2:** Total (Poly)phenols and Phenolic Catabolites from Raw and *Sous-Vide*-Cooked Red Cardoon before and after Simulated Oral GI Digestion *In Vitro*, after Subsequent Dialysis and during a Simulated Colonic Fermentation *In Vitro* (0, 8, 24, and 48 h), as Analyzed by HPLC–MS/MS^a^

(poly)phenol	red cardoon							
	nondigested	digested	digested and dialyzed	0 h fermented	4 h fermented	8 h fermented	24 h fermented	48 h fermented
**Phenolic Acids**								
*Monocaffeoylquinic Acids* (CQAs) *and Derivatives*								
raw	5707.33	55.27 (1%)	41.04 (0.7%)	29.10 (0.5%)	15.60 (0.3%)	16.73 (0.3%)	0.78 (<0.1%)	0.19 (<0.1%)
*Sous vide*	6402.44	4393.86 (68.6%)	3318.66 (51.8%)	2035.07 (31.8%)	1560.62 (24.4%)	37.56 (0.6%)	6.82 (0.1%)	0.82 (<0.1%)
*Dicaffeoylquinic Acids* (diCQAs) *and Derivatives*								
raw	7308.38	13.09 (0.2%)	8.00 (0.1%)	3.51 (<0.1%)	1.11 (<0.1%)	1.01 (<0.1%)	<LOQ	<LOQ
*Sous vide*	6464.06	3295.71 (51%)	1602.48 (24.8%)	794.63 (12.3%)	760.73 (11.8%)	251.38 (3.9%)	6.43 (0.1%)	2.33 (<0.1%)
*Other Hydroxycinnamic Acids*								
raw	7.40	1.01 (13.6%)	1.16 (15.7%)	0.49 (6.7%)	2.92 (39.4%)	<LOQ	<LOQ	<LOQ
*Sous vide*	72.09	8.20 (11.4%)	1.60 (2.2%)	7.39 (10.3%)	49.27 (68.4%)	1.30 (1.8%)	2.76	2.02
*Total phenolic Acids*								
raw	13,023.11	69.37 (0.5%)	50.21 (0.4%)	33.10 (0.3%)	19.63 (0.2%)	17.74 (0.1%)	0.78 (<0.1%)	0.19 (<0.1%)
*Sous vide*	12,938.58	7697.77 (59.5%)	4922.74 (38%)	2837.09 (21.9%)	2370.62 (18.3%)	290.24 (2.2%)	16.01 (0.1%)	5.17 (<0.1%)
**Flavonoids**								
*Apigenin Derivatives*								
raw	3.56	2.68 (75.3%)	0.74 (20.8%)	<LOQ	<LOQ	0.98 (27.5%)	<LOQ	<LOQ
*Sous vide*	20.59	14.13 (68.6%)	5.98 (29%)	4.20 (20.4%)	4.14 (20.1%)	3.65 (17.8%)	<LOQ	<LOQ
*Luteolin Derivatives*								
raw	119.81	20.77 (17.3%)	9.77 (8.2%)	7.23 (6%)	5.32 (4.4%)	4.24 (3.5%)	0.22 (0.2%)	0.18 (0.2%)
*Sous vide*	158.58	135.60 (85.5%)	69.08 (43.6%)	50.08 (31.6%)	28.55 (18%)	26.04 (16.4%)	7.41 (4.7%)	5.64 (3.6%)
*Quercetin Derivatives*								
raw	2.72	<LOQ	0.00	0.00	0.00	<LOQ	0.00	0.00
*Sous vide*	6.63	3.14 (47.3%)	1.42 (21.4%)	<LOQ	<LOQ	1.65 (24.9%)	<LOQ	0.00
**Total Flavonoids**								
raw	126.09	23.45 (18.6%)	10.51 (8.3%)	7.23 (5.7%)	5.32 (4.2%)	5.22 (4.1%)	0.22 (0.2%)	0.18 (0.1%)
*Sous vide*	197.70	161.21 (82.3%)	84.39 (41.2%)	54.28 (29.2%)	32.68 (17.6%)	31.34 (16.9%)	7.41 (4%)	5.64 (3%)
**Total (P**oly)phenols								
raw	13,149.20	92.82 (0.7%)	60.72 (0.5%)	40.33 (0.3%)	24.95 (0.2%)	22.96 (0.2%)	1.00 (<0.1%)	0.37 (<0.1%)
*Sous vide*	13,136.28	7858.98 (59.8%)	5007.13 (38.1%)	2891.37 (22%)	2403.30 (18.3%)	321.58 (2.5%)	23.42 (0.2%)	10.81 (0.1%)
**Phenolic Catabolites**								
raw	0.00	0.00	0.00	67.05	73.95	100.84	238.22	863.13
*Sous vide*	0.00	0.00	0.00	72.08	123.73	584.65	1918.16	5899.00

a Results are expressed as total μg (poly)phenolic compound per g red cardoon sample dry matter. Total (poly)phenol bioaccessibility (%) is included in brackets. <LOQ, below the limit of quantification.

Despite raw and *sous-vide*-cooked red cardoon having a similar total content of (poly)phenols (13.15 and 13.14 mg/g dm respectively), differences in the content or in the individual (poly)phenol profile were found. The most abundant (poly)phenols were 5-CQA (29.3–41.8% of total polyphenols) and 1,5-diCQA (20.7–39.2% of total polyphenols) in both cardoon samples, along with 3,5-diCQA (13.0% of total polyphenols) in raw cardoon, and 1,3-diCQA (18.2%) in the *sous vide* one. In addition, undigested raw cardoon showed a higher content of 5-CQA (1.4-fold), 1,5-diCQA (3-fold), 3,5-diCQA (1.9-fold), and succinyldiCQA I (2-fold) and II (13.7-fold) than *sous vide* cardoon. On the contrary, 11 (poly)phenols (i.e., CQA derivatives I–III, 3,4-diCQA, diCQA glucoside I, ferulic and isoferulic acids, apigenin, vicenin-2, luteolin, and hesperedin), which were only found in trace amounts or not detected in the raw cardoon, could be quantified in the *sous vide* one.

After an *in vitro* simulated oral-GI digestion, the content of individual (poly)phenols substantially decreased in both samples (Table 1), reaching a bioaccessibility of 0.7% (or 0.5% after dialysis) in digested raw cardoon, whereas 59.8% (or 38.1% after dialysis) of (poly)phenols still remained bioaccessible in digested *sous-vide*-cooked cardoon (Table 2).

During the 48 h *in vitro* simulated colonic fermentation, (poly)phenol bioaccessibility of raw and *sous vide* cardoon gradually decreased, while 9 catabolites were detected at different fermentation time points (Table 1). In addition, flavonoid aglycones such as luteolin and apigenin, as well as low-molecular-weight hydroxycinnamic acids, such as caffeic acid, increased during 4–8 h of fermentation but decreased upon further fermentation. Both phenolic acids and flavonoids were mainly catabolized during the first 8 h of colonic fermentation, remaining bioaccessible for 0.1–2.2% of phenolic acids and 4.1–16.9% of flavonoids (Table 2). During the first 4 h of colonic fermentation, five catabolites were detected [i.e., 3-hydroxyphenylacetic, 2,5-dihydroxybenzoic, protocatechuic, 3-(3-hydroxyphenyl)propionic and dihydrocaffeic acids], and their contents gradually increased throughout the 48 h of colonic fermentations, except for dihydrocaffeic acid whose content diminished after 8 h (Table 1). The catabolite 4-hydroxybenzoic acid was produced after 8 h of fermentation of both samples; and phenylacetic acid and 1,2-dihydroxybenzene were produced after 24 h. Small amounts of 3-hydroxybenzoic acid were quantified at 48 h of fermentation of both raw and *sous-vide*-cooked cardoon. The highest total content of catabolites occurred after 48 h of fermentation of both raw and *sous-vide*-cooked red cardoon (863.13 and 5899 μg/g dm, respectively) (Table 2). Finally, the total content of catabolites was higher in *sous vide* than in raw cardoon at different time points (1.7-fold at 4 h, 5.8-fold at 8 h, 8-fold at 24 h, and 6.8-fold at 48 h) (Table 2), mainly due to the increase of the most abundant catabolites [3-hydroxyphenylacetic and 3-(3-hydroxyphenyl)propionic acids] (Table 1).

### *In Vitro* Anti-inflammatory Activity

**Figure 1:**
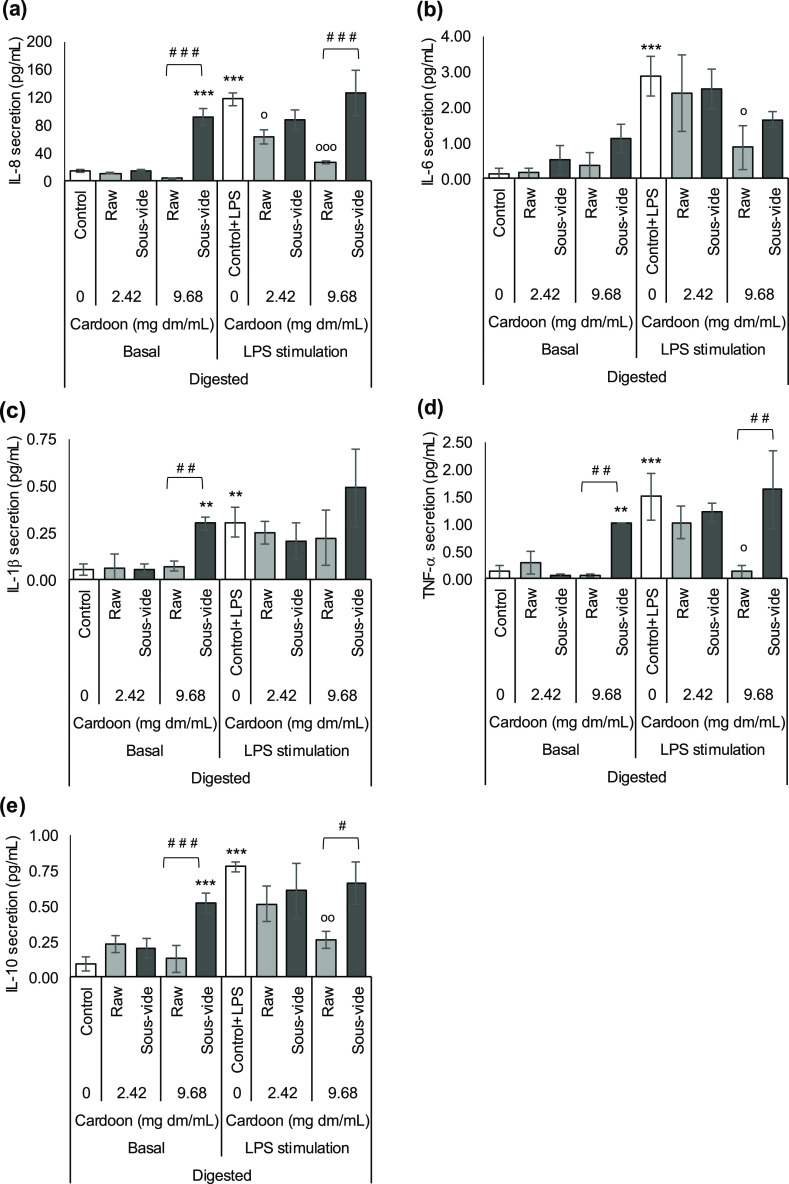
Impact of the digested raw and *sous vide* red cardoon on basal and LPS-induced secretion of cytokines (a) IL-8, (b) IL-6, (c) IL-1β, (d) TNF-α, and (e) IL-10 in differentiated Caco-2 cells (Luminex multiplex assay). Cells were pretreated with supernatants of the digested cardoon samples for 1 h, and treatment was continued for further 48 h in the absence or presence of 5 μg/mL LPS. Results are expressed as the mean of picograms of cytokine secretion per milliliter (pg/mL) ± SD (*n* = 3 experiments). *p < 0.05, **p < 0.01, and ***p < 0.001 indicate significantly different from control (untreated cells). ^o^*p* < 0.05, ^oo^*p* < 0.01, and ^ooo^*p* < 0.001 indicate significantly different from control + LPS. ^#^*p* < 0.05, ^##^*p* < 0.01, and ^###^*p* < 0.001 indicate significant differences among raw and *sous vide* cardoon.

The anti-inflammatory activity in the small intestine was evaluated by measuring the impact of GI-digested raw and *sous vide* cardoon on basal and LPS-stimulated cytokine secretion in differentiated Caco-2 cells. GI-digested raw red cardoon neither significantly modified the basal secretion of the tested cytokines (IL-8, IL-6, IL-1β, TNF-α, and IL-10) in cells at any of the concentrations (i.e., 2.42 and 9.68 mg dm/mL), as compared to the control (untreated cells) (Figure 1a–e), nor did the digested *sous-vide*-cooked cardoon induced significant differences in the basal secretion of the tested cytokines at 2.42 mg dm/mL but caused a significant increase of IL-8 (*p* < 0.001), IL-1β (*p* = 0.001), TNF-α (*p* = 0.002), and IL-10 (*p* < 0.001) at 9.68 mg dm/mL, as compared to control. In addition, the increased secretion of IL-1β and TNF-α induced by digested *sous-vide*-cooked cardoon at 9.68 mg dm/mL was not significantly different to that caused by LPS.

Differentiated Caco-2 cells stimulated with 5 μg/mL LPS for 48 h (called “control + LPS”) showed a significant rise in IL-8, IL-6, TNF-α, IL-10 (*p* < 0.001), and IL-1β (*p* = 0.001) secretions (Figure 1a–e). Control + LPS was used as the control to assess the impact of GI-digested cardoon on the LPS-induced secretion of cytokines. At 2.42 mg dm/mL, digested raw cardoon significantly decreased the LPS-induced secretion of IL-8 (*p* = 0.020), whereas at 9.68 mg dm/mL it induced a significant reduction in the LPS-induced secretion of IL-8 (*p* < 0.001), IL-6 (*p* = 0.026), TNF-α (*p* = 0.014), and IL-10 (*p* = 0.004) to levels that were not significantly different from the control without LPS stimulation. On the contrary, digested *sous-vide*-cooked red cardoon did not significantly change the LPS-induced secretion of IL-8, IL-6, IL-1β, TNF-α, or IL-10 at the tested concentrations.

**Figure 2:**
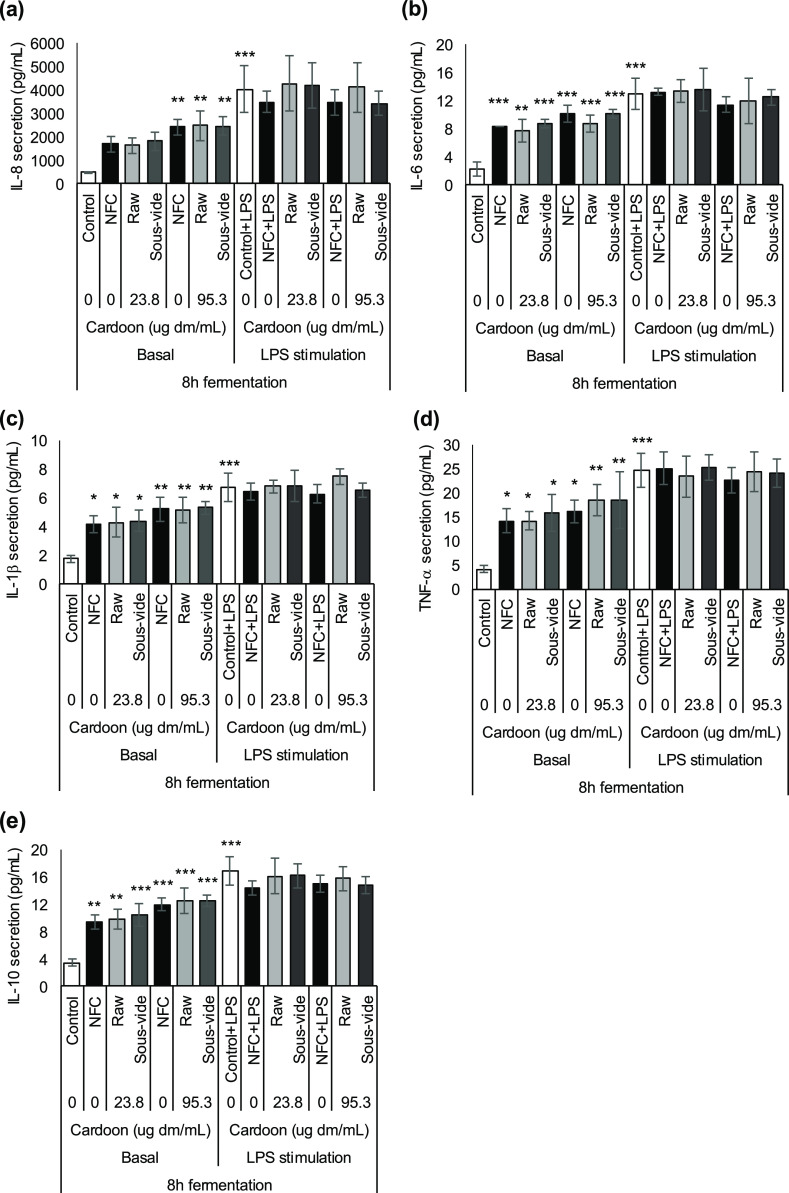
Impact of 8 h fermented raw and *sous vide* red cardoon on basal and LPS-induced secretion of cytokines (a) IL-8, (b) IL-6, (c) IL-1β, (d) TNF-α, and (e) IL-10 in HT-29 cells (Luminex multiplex assay). Cells were pretreated with supernatants from 8 h fermentation of cardoon samples or NFC at 0.25 or 1% v/v for 1 h, and treatment was continued for further 48 h in the absence or presence of 0.1 μg/mL LPS. Results are expressed as the mean of picograms of cytokine secretion per milliliter (pg/mL) ± SD (*n* = 3 experiments). *p < 0.05, **p < 0.01, and ***p < 0.001 indicate significantly different from control (untreated cells). Lack of asterisk indicates nonsignificant differences (*p* ≥ 0.05) (both in comparison with control and NFC values, either with or without LPS stimulation, and between raw and *sous vide* cardoon).

**Figure 3:**
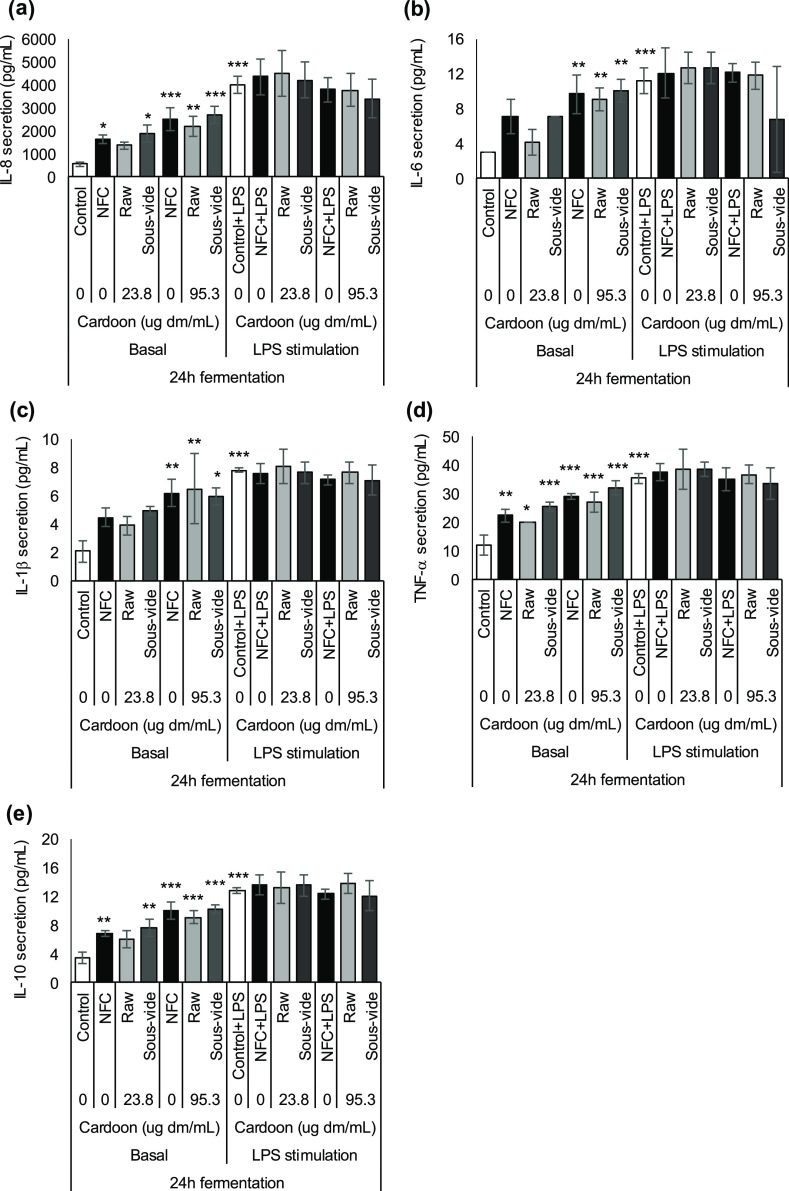
Impact of 24 h fermented raw and *sous vide* red cardoon on the basal and LPS-induced secretion of cytokines (a) IL-8, (b) IL-6, (c) IL-1β, (d) TNF-α, and (e) IL-10 in HT-29 cells (Luminex multiplex assay). Cells were pretreated with supernatants from 24 h of fermentation of cardoon samples or NFC at 0.25 or 1% v/v for 1 h, and treatment was continued for further 48 h in the absence or presence of 0.1 μg/mL LPS. Results are expressed as the mean of picograms of cytokine secretion per milliliter (pg/mL) ± SD (*n* = 3 experiments). *p < 0.05, **p < 0.01, and ***p < 0.001 indicate significantly different from control (untreated cells). Lack of asterisk indicates nonsignificant differences (*p* ≥ 0.05) (both in comparison with control and NFC values, either with or without LPS stimulation, and between raw and *sous vide* cardoon).

The anti-inflammatory activity in the colon was evaluated by measuring the impact of GI-digested and colonic-fermented raw and *sous vide* cardoon on basal and LPS-stimulated cytokine secretion in HT-29 cells. Untreated HT-29 cells were used as the blank control (called “control”), and cells incubated with supernatants from 8 or 24 h NFC were used as a carrier control (called “NFC”) for the assessment of the anti-inflammatory activity of 8 and 24 h colonic fermented raw and *sous-vide*-cooked red cardoon. NFC of either 8 h (Figure 2) or 24 h (Figure 3) colonic fermentation generally induced a significant stimulation of the basal cytokine secretion in the HT-29 cells as compared to the control (*p* < 0.05). Neither 8 nor 24 h fermented raw and *sous-vide*-cooked cardoon changed the cytokine secretion stimulated by NFC at the tested concentrations (23.8 and 95.3 μg dm/mL).

HT-29 cells treated with 0.1 μg/mL of LPS for 48 h (called “control + LPS”) induced a highly significant increase (*p* < 0.001) of all the tested cytokines with respect to the control (Figures 2 and 3). Co-treatment of NFC from either 8 h (Figure 2) or 24 h (Figure 3) colonic fermentation and LPS (NFC + LPS) showed no differences in IL-8, IL-6, IL-1β, TNF-α, and IL-10 secretions with respect to control + LPS. Neither raw nor *sous-vide*-cooked red cardoon, either 8 or 24 h colonic fermented, showed significant differences in the cytokine secretion with respect to control + LPS or NFC + LPS at the tested concentrations (23.8 and 95.3 μg dm/mL). No significant differences were found between fermented raw and *sous vide* cardoon in their impact on the basal and LPS-induced secretion of the tested cytokines in HT-29 cells (Figures 2 and 3).

### *In Vitro* Prebiotic Activity

#### Impact on the Growth of Selected Bacterial Populations of Gut Microbiota

**Figure 4:**
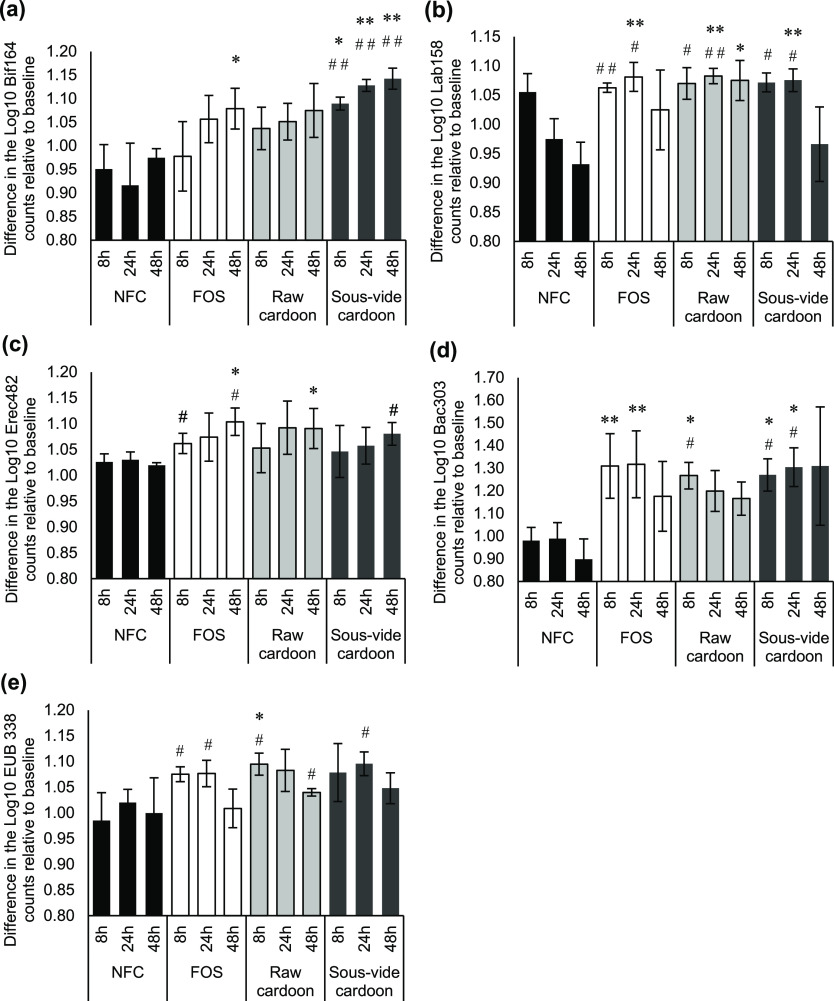
Impact of raw and *sous vide* red cardoon on the growth of specific bacterial populations (a) *Bifidobacterium* spp. (Bif164), (b) *Lactobacillus*/*Enterococcus* spp. (Lab158), (c) *E. rectale*–*C. coccoides* group (Erec482), (d) *Bacteroides*–*Prevotella* group (Bac303), and (e) total bacteria (EUB 338), at 8, 24, or 48 h of *in vitro* batch-cultured colonic fermentation, as analyzed by FISH. NFC and prebiotic FOS are included. Results are expressed as the mean of the difference in the log10 bacterial counts of each specific bacteria relative to the baseline (0 h) ± SD (*n* = 3 experiments). *p < 0.05, **p < 0.01, and ***p < 0.001 indicate significantly different from NFC at the same time point. #*p* < 0.05 and ##*p* < 0.01 indicate significantly different from the baseline within the same treatment.

The fermentation of s*ous-vide*-cooked cardoon significantly (*p* < 0.05) enhanced the growth of *Bifidobacterium* spp. after 8, 24, and 48 h of fermentation compared to the baseline and NFC (Figure 4a). This bifidogenic effect was similar to that observed with the positive control FOS at the end of the fermentation (1.08 ± 0.04 and 1.14 ± 0.02 log10 bacterial counts of bifidobacteria after 48 h of FOS and *sous-vide*-cooked cardoon fermentations, respectively, relative to the baseline). Raw cardoon also induced the stimulation of bifidobacteria, which was close to reaching statistical significance compared to NFC at 48 h (*p* = 0.052) (1.08 ± 0.06 and 0.97 ± 0.02 log10 bacterial counts of bifidobacteria after 48 h of raw cooked cardoon fermentations and NFC, respectively, relative to the baseline). Moreover, numbers of *Lactobacillus*/*Enterococcus* were also significantly increased after 24 h of fermentation with raw and *sous-vide*-cooked cardoon (*p* = 0.003 and *p* = 0.004, respectively) and FOS (*p* = 0.003). This stimulation still persisted after 48 h of fermentation with raw cardoon (*p* = 0.043) (Figure 4b). Increases of *E. rectale*–*C. coccoides* were also detected with raw cardoon and FOS fermentations at 48 h compared to NFC (*p* = 0.040 and 0.017) (Figure 4c). A significant stimulation of *Bacteroides*–*Prevotella* spp. was observed at 8 h of raw cardoon fermentation (*p* = 0.019) and at 8 and 24 h of *sous-vide*-cooked cardoon (*p* = 0.018 and 0.023) and FOS (*p* = 0.009 and 0.019) fermentations compared with NFC (Figure 4d). *Bacteroides*–*Prevotella* spp. showed the highest growth during fermentation when compared with the other tested populations of gut microbiota. There was no significant change in the total bacterial numbers with *sous-vide*-cooked cardoon and FOS, while a significant increase was observed at 8 h of raw cardoon fermentation (*p* = 0.046) (Figure 4e).

#### Impact on Lactic Acid and SCFA Production by Gut Microbiota

Significant increases of acetate were detected during the fermentation of raw and *sous-vide*-cooked cardoon compared to the baseline (0 h) and NFC (*p* < 0.05) (Table 3). The highest concentration of acetate was detected at 48 h of fermentation with raw cardoon and FOS and at 24 and 48 h of fermentation with *sous-vide*-cooked cardoon. Propionate was the second more abundant SCFA produced during the fermentation of FOS and cardoon samples (Table 3). Similar to acetate, propionate production showed an increasing trend during fermentation but the highest increase occurred from 8 h to 24 h of fermentation of cardoon samples. The propionate concentration from raw and *sous-vide*-cooked cardoon fermentations was not significantly higher than that in NFC at any time point, and it reached a statistical significance after 48 h of FOS fermentation (*p* = 0.003). Although the production of butyrate was lower than acetate and propionate, a slight but significant increase in the butyrate concentration was observed at 24 and 48 h of fermentation with raw and *sous-vide*-cooked cardoon compared to the baseline (0 h) (*p* < 0.05). However, this increase did not reach a statistical significance compared to NFC. The highest amount of butyrate was detected with FOS after 48 h of fermentation and this increase was significant compared to NFC (*p* = 0.014). The lactate concentration was very low in all the samples and did not reach a statistical significance compared to NFC (Table 3). Concentrations of isobutyric and isovaleric acids were either below the detection limit or, in many cases, were detected only in a single donor (data not shown). The concentration of total SCFAs increased significantly (*p* < 0.05) at 8, 24, and 48 h of FOS fermentation, and in the case of raw and *sous-vide*-cooked cardoon fermentation at 48 h compared to NFC (*p* < 0.05). The highest concentrations of total SCFAs were found at 48 h of fermentation of FOS and cardoon samples, mainly due to acetate production but also propionate production. No significant differences were found between raw and *sous-vide*-cooked cardoon regarding their impact on lactic acid and SCFA production.

**Table 3:** Impact of Raw and *Sous-Vide*-Cooked Red Cardoon on the Production of Major SCFAs (Acetic, Propionic, and Butyric Acids) and Lactic Acid at Different Time Points (0, 8, 24, and 48 h) of *In Vitro* Colonic Fermentation, as Analyzed by GC-FID^a^

SCFA	NFC	FOS	raw cardoon	*s*ous vide-cooked cardoon
Acetate				
0 h	0.29 ± 0.29	0.26 ± 0.26	1.24 ± 0.07*	1.59 ± 0.05**
8 h	4.88 ± 1.48	17.68 ± 1.51## **	16.65 ± 1.38## **	19.91 ± 0.64## ***
24 h	6.96 ± 4.49	32.28 ± 3.63# **	25.27 ± 3.89## *	33.41 ± 2.84## **
48 h	8.67 ± 4.68	38.77 ± 2.69## **	34.22 ± 2.92# **	33.76 ± 4.69# **
Propionate				
0 h	nd	nd	nd	Nd
8 h	1.02 ± 0.52	5.40 ± 4.06	2.75 ± 0.93	2.64 ± 0.80
24 h	1.61 ± 0.92	14.48 ± 7.13	9.80 ± 3.25	12.92 ± 4.72
48 h	2.14 ± 1.00	26.80 ± 2.39## **	11.65 ± 2.89	12.81 ± 5.28
Butyrate				
0 h	nd	nd	nd	Nd
8 h	0.36 ± 0.35	0.87 ± 0.44	0.10 ± 0.10	0.10 ± 0.10
24 h	0.52 ± 0.52	3.94 ± 1.76	1.74 ± 0.46#	1.35 ± 0.15#
48 h	1.05 ± 0.61	6.83 ± 1.81*	2.06 ± 0.36#	1.70 ± 0.36#
Lactate				
0 h	nd	nd	nd	Nd
8 h	2.17 ± 2.16	3.01 ± 0.52#	3.42 ± 1.05	4.71 ± 1.92
24 h	0.01 ± 0.00	6.76 ± 5.18	2.10 ± 2.10	2.30 ± 2.30
48 h	nd	nd	1.60 ± 1.60	2.07 ± 2.07
Total				
0 h	0.29 ± 0.29	0.26 ± 0.26	1.24 ± 0.07*	1.59 ± 0.05**
8 h	8.43 ± 1.87#	26.96 ± 5.53*	22.92 ± 2.98#	27.36 ± 2.52## *
24 h	9.09 ± 5.88	57.46 ± 14.98# *	38.92 ± 6.82#	49.98 ± 7.61#
48 h	11.86 ± 6.29	72.40 ± 4.30**	49.53 ± 5.19# *	50.33 ± 9.33# *

a The effect of NFC and prebiotic FOS is also included. Results are expressed as mean mM of lactic acid or SCFA of three independent fermentations with fecal samples from three different donors ±standard error of the mean (SEM). #*p* < 0.05 and ##*p* < 0.01, significantly different from the 0 h value within the same treatment. *p < 0.05, **p < 0.01, and ***p < 0.001, significantly different from NFC at the same time point. nd, not detected.

## Discussion

The application of culinary heat treatments to (poly)phenolic-rich plant foods, such as cultivated cardoon stalks, induces wall and cell ruptures and consequently the release of those (poly)phenols bound to the food matrix.^3,20^ However, some (poly)phenols remained linked to other macromolecules, such as dietary fiber during boiling,^21,22^ or are included into melanoidin structures when formed at high temperatures.^23,24^ Moreover, the inactivation of polyphenol oxidases by heat inhibits (poly)phenol degradation, and the use of little water and vacuum bags in the *sous vide* cooking avoids the (poly)phenols leaching into the water. All these reasons may explain the similar total content of (poly)phenols in raw and *sous-vide*-cooked red cardoon. After GI digestion, (poly)phenols were almost totally degraded by the digestive enzymes and conditions in raw red cardoon, whereas they remained substantially bioaccessible in *sous-vide*-cooked red cardoon. A similar behavior in (poly)phenol bioaccessibility has been observed in cooked white cardoon,^3^ blanched^25,26^ and *sous-vide*-cooked globe artichoke,^4^ which are rich in phenolic acids, mainly CQAs. However, less degradation of (poly)phenols, particularly phenolic acids, was shown in raw *Opuntia* cactus cladodes, which is rich in pectins, mucilages, and other dietary fibers.^22^ Then, the hydration of the dietary fiber during boiling may favor the retention of phenolic acids into macromolecules, inducing less degradation by digestive enzymes and conditions. This may also explain the positive effect of *sous vide* heat treatment on the bioaccessibility of red cardoon (poly)phenols and particularly CQAs.

The *in vitro* anti-inflammatory activity of red cardoon stalks, in two culinary ways of consumption (raw and *sous-vide*-cooked), was assessed at both the small intestine (differentiated Caco-2 cells) and colon (HT-29 cells) level. LPS was used to induce a proinflammatory phenotype in both cell lines and thereby to evaluate the ability of red cardoon to protect against LPS-induced secretion of IL-8, IL-6, IL-1β, TNF-α, and IL-10. No previous studies that evaluate the *in vitro* immunomodulatory activity of cardoon stalks, whether undigested, GI-digested, or colonic-fermented have been found.

The bioaccessible fraction of GI-digested raw red cardoon, but surprisingly not the *sous-vide*-cooked one, exerted *in vitro* anti-inflammatory capacity in the human enterocyte-like cell line Caco-2 (Figure 1). This inflammatory protection was stronger at the highest concentration tested (9.68 mg dm/mL), which significantly (*p* < 0.05) counteracted the LPS-induced secretion of IL-8, IL-6, TNF-α, and IL-10. Contrarily, the bioaccessible fraction of digested *sous-vide*-cooked cardoon showed strong proinflammatory effects at the highest tested concentration (9.68 mg dm/mL) in Caco-2 cells in the absence of LPS stimulation (Figure 1). The most abundant (poly)phenol of digested red cardoon samples (5-CQA) was able to inhibit TNF-α- and H_2_O_2_-induced IL-8 secretions in a dose-dependent manner (0.5–2 mM) in differentiated Caco-2 cells.^27^ However, both anti- and proinflammatory effects have been reported in (poly)phenols, and their biological effects depend on the state of the target cells (e.g., resting vs activated), cell ontogeny and pathological conditions (e.g., normal vs cancer cells or macrophages), and the (poly)phenol concentration, exposure times, and pharmacokinetics.^28^ The higher bioaccessibility of (poly)phenols (and potentially other phytochemicals not identified in the current study) in digested *sous-vide*-cooked red cardoon might have led to an increased absorption of these compounds in the enterocyte-like cells,^29^ causing a proinflammatory response at high concentrations. This study corroborates that the consumption of higher amounts of (poly)phenols in a diet does not necessarily increase inflammatory protection.^28^ Considering a consumption of 150 g of cardoon, the tested concentrations (i.e., 2.42 and 9.68 mg cardoon dm/mL) would correspond to around 4.7 and 1.2 L of fluid in the small intestine, respectively. The small intestine receives about 9.3 L of fluid each day and absorbs around 8.3 L during the day,^30^ so the tested concentrations were considered physiologically achievable. It would also be interesting to assess whether, in a context of a meal in which other non-phenolic-rich foods are included, lower concentrations of (poly)phenols from digested *sous-vide*-cooked cardoon could exert anti-inflammatory activity at the small intestine level. Also, further studies on the intestine inflammatory protection of other potential bioactive compounds different than (poly)phenols present in raw red cardoon would be of interest.

In a colon cell model (HT-29), after colonic fermentation, the high proinflammatory activity of NFC at the baseline (*p* < 0.05) may be related to the presence of some harmful compounds or metabolites derived from the gut microbiota activity of volunteers, which is in agreement with the highly reported adverse effects of fecal water on intestinal cells (cytotoxicity, mutagenicity, and genotoxicity).^31,32^ Gut microbiota action induced the formation of phenolic catabolites, nine of which were identified and quantified in the bioaccessible fractions of colonic fermented raw and *sous-vide*-cooked red cardoon. In the study reported by Juániz et al., 3-(3-hydroxyphenyl)propionic acid was the most abundant catabolite produced during white cardoon stalks *in vitro* fermentation,^3^ whereas in the current study that catabolite was the second most abundant one after 3-hydroxyphenylacetic acid. Juániz et al. did not identify 3-hydroxyphenylacetic acid,^3^ which might come from the α-oxidation of 3-(3′-hydroxyphenyl)propionic acid by gut microbiota.^33^ According to the proposed catabolic pathway for CQA degradation by Ludwig et al., red cardoon (poly)phenols, followed a major pathway that involved the formation of caffeic acid, followed by dihydrocaffeic acid, 3-(3′-hydroxyphenyl)propionic acid and 3-hydroxyphenylacetic acid as the final compounds identified.^33^ Despite the fact that *sous-vide*-cooked cardoon had a higher total content of (poly)phenols and catabolites (7–8-fold) than the raw one (Table 2), no response was observed in the secretion of cytokines induced by the NFC or LPS (Figures 2 and 3). The tested concentrations of colonic fermented cardoon samples (23.8 and 95.3 μg dm cardoon/mL) were around 100-fold lower than those of the digested vegetable due to cytotoxicity issues and are equivalent to an intake of 312 and 1250 mg of cardoon, respectively, for a fluid volume of 1 L in the colon.^30^ These data raise the question of whether higher concentrations of colonic fermented red cardoon (raw and *sous-vide*-cooked) may have the ability to modulate cytokine secretion in HT-29 cells, as digested cardoon did in differentiated Caco-2 cells.

To the best of the authors’ knowledge, this study also reports for the first time the impact of red cardoon (raw and *sous-vide*-cooked) on gut microbiota composition and SCFA production. Red cardoon showed a potential prebiotic effect on the human microbiota comparable to the well-established prebiotic FOS used as a reference control. Raw and *sous-vide*-cooked cardoon led to a beneficial shift of the microbiota composition by increasing health-promoting bacteria such as *Bifidobacterium* spp. and *Lactobacillus*/*Enterococcus* (Figure 4) as well as stimulating the production of SCFAs (Table 3). Moreover, increases in other bacterial populations such as *Bacteroides*–*Prevotella* spp. and *E. rectale*–*C. coccoides* were detected (Figure 4). *Bacteroides*–*Prevotella* spp. (8.5%) and *E. rectale*–*C. coccoides* (28%) are two of the most predominant groups in the human fecal microbiota.^34^ Some species of these groups are related with some pathological conditions (*Bacteroides* are increased in IBD and some species of *Clostridium* are pathogenic),^9^ but others may be considered potentially beneficial due to their saccharolytic metabolism that results in the production of SCFAs, which are known to have beneficial effects on the host health.^35^ Hence, the *E. rectale*–*C. coccoides* group includes many acetate- and/or lactate-converting butyrate producers,^36^ and an increase in the production of butyric acid was observed with raw cardoon, *sous-vide*-cooked cardoon, and FOS fermentations at 24 and 48 h, although it did not reach statistical significance due to great variability in the butyrate production among volunteers (Table 3). In addition, *Bacteroides*/*Prevotella* mainly produce acetic, succinic, and propionic acids,^37^ and a significantly high production of propionic acid with the raw and *sous-vide*-cooked red cardoon fermentations was observed at 24 and 48 h (*p* < 0.05), as compared to the baseline. Acetic acid was the main SCFA produced by both raw and *sous-vide*-cooked cardoon fermentations. This correlates with the fact that acetic acid is the most abundant SCFA produced, representing around 60–75% of the total SCFA in feces, as acetate synthesis is a widespread ability in the human gut microbiota.^37^

Overall, slight differences were observed between the raw and the *sous-vide*-cooked vegetable. For instance, *sous-vide*-cooked cardoon as the substrate had a higher bifidogenic effect and longer effect in the stimulation of *Bacteroides*–*Prevotella* than raw cardoon. The application of culinary treatments has been shown to impact not only bioactive compounds such as (poly)phenols but also dietary fibers. Culinary treatments on vegetables cause the breakdown of macrostructures and mesostructures and increase the solubility of dietary fibers, making them more digestible.^36^ Thus, the differences between raw and *sous-vide*-cooked red cardoon on the stimulation of different bacterial populations may be related to their different contents of soluble fibers and (poly)phenols (and potentially other bioactive compounds). There is evidence about the ability of both fibers and (poly)phenols to positively modulate the composition and function of gut microbiota.^9,10^ Specifically, dietary fiber (mainly inulin-type fructans) obtained from artichoke, a vegetable that belongs to the same genus as cardoon, showed a prebiotic effect by stimulating the growth of different strains of *Lactobacillus* and *Bifidobacterium* spp.^38,39^ In addition, the coffee sample with high levels of chlorogenic acids [the main type of (poly)phenols in coffee that occurs in cardoon] induced a significant increase of *Bifidobacterium* spp. growth, and an equivalent quantity of chlorogenic acids alone induced the same effects along with an increase of the *C. coccoides*–*E. rectale* group.^40^ According to this, the stimulation of *Bifidobacterium* spp. by red cardoon may be due to the action of both fibers and (poly)phenols.

In summary, the application of a culinary treatment, and the GI digestion and gut microbiota action, following a realistic intake *in vitro* approach affects the (poly)phenol bioaccessibility and bioactivity of red cardoon stalks in the GI tract. Digested raw red cardoon showed anti-inflammatory activity against LPS-induced secretion of IL-8, IL-1β, TNF-α, and IL-10, while digested *sous vide* red cardoon with a higher content of (poly)phenols showed proinflammatory effects in enterocyte-like cells. However, colonic fermented raw and *sous-vide*-cooked cardoon did not show anti-inflammatory activity in HT-29 cells but both induced a beneficial effect on gut microbiota by stimulating *Bifidobacterium* spp. (especially *sous vide* cardoon) and *Lactobacillus*/*Enterococcus* spp. growth and by increasing the production of acetic acid mainly. Therefore, this study provides insights into mechanisms through which red cardoon stalk consumption might influence digestive health and provides evidence for further investigation in health-induced benefits of *Cynara* vegetables.

## Supporting Information Available

MS characteristics of (poly)phenolic compounds identified in this study and cytotoxicity (MTT assay) of raw and *sous-vide*-cooked red cardoon after GI digestion in differentiated Caco-2 cells and after 8 and 24 h of colonic fermentation in HT-29 cells, with and without LPS stimulation (PDF)

## Abbreviations

CQAs: caffeoylquinic acids
diCQAs: dicaffeoylquinic acids
FISH: fluorescence *in situ* hybridization
FOS: fructo-oligosaccharide
GC-FID: gas chromatography with flame ionization detection
GI: castrointestinal
HPLC–MS/MS: high-performance liquid chromatography with tandem mass spectrometry
IL: interleukin
SCFAs: short-chain fatty acids systemic chronic inflammation
SCI: systemic chronic inflammation
LOQ: limit of quantification
MTT: 3-(4,5-dimethythiazol-2-yl)-2,5-diphenyltetrazolium bromide
NFC: negative fermentation control
TNF: tumor necrosis factor
