# Supplementary material for: Raw and Sous-Vide-Cooked Red Cardoon Stalks (Cynara cardunculus L. var. altilis DC): (Poly)phenol Bioaccessibility, Anti-inflammatory Activity in the Gastrointestinal Tract, and Prebiotic Activity
Source: J Agric Food Chem. 2021 Aug 4;69(32):9270–86. doi: 10.1021/acs.jafc.1c03014 (PMC8389834; doi:10.1021/acs.jafc.1c03014)

SUPPLEMENTARY DATA

TITLE: Raw and *sous-vide* cooked red cardoon stalks (*Cynara cardunculus* L. var. *altilis* DC): (poly)phenol bioaccessibility, anti-inflammatory activity in the gastrointestinal tract, and prebiotic activity

AUTHORS: Estíbaliz Huarte[1], Gessica Serra[2], Andrea Monteagudo-Mera[2], Jeremy Spencer[2], Concepción Cid[1,3], and María-Paz de Peña[1,3]*

[1] Departamento de Ciencias de la Alimentación y Fisiología, Facultad de Farmacia y Nutrición, Universidad de Navarra C/ Irunlarrea 1, 31008 Pamplona, Spain

[2] Department of Food and Nutritional Sciences, University of Reading, Whiteknights, PO Box 226, Reading RG6 6AP, UK

[3] IdiSNA, Navarra Institute for Health Research C/ Irunlarrea 1, 31008 Pamplona, Spain

* Corresponding author: María-Paz de Peña. Tel: +34 948 425600 (806580); Fax: +34 948 425740. E-mail address: mpdepena@unav.es

Table S1. Mass spectrometric characteristics of (poly)phenolic compounds identified in this study.

Nº	(Poly)phenolic compound	R_t (min)	$[M–H]^-$ *(m/z)*	Fragment ions *(m/z)*
**Phenolic acids**				
Monocaffeoylquinic acids (CQAs) and derivatives				
1	1–CQA	2.11	353	191 (Q), 179
2	3–CQA [a]	3.24	353	191 (Q), 179
3	4–CQA [a]	6.73	353	173 (Q), 179
4	5–CQA [a]	5.85	353	191 (Q), 179
5	CQA derivative I	10.47	631	353 (Q), 191
6	CQA derivative II	13.08	793	353 (Q), 191
7	CQA derivative III	15.10	793	353 (Q), 191
Dicaffeoylquinic acids (diCQAs) and derivatives				
8	1,3–diCQA [a]	8.69	515	353 (Q), 191, 179
9	1,5–diCQA [a]	12.27	515	191 (Q), 353
10	3,5–diCQA [a]	12.39	515	353 (Q), 191
11	3,4–diCQA [a]	11.88	515	353 (Q), 335, 299
12	4,5–diCQA [a]	13.38	515	353 (Q), 173
13	DiCQA glucoside I	10.22	677	191 (Q), 323
14	DiCQA glucoside II	10.32	677	323 (Q), 515
15	SuccinyldiCQA I	13.40	615	453 (Q), 353, 191
16	SuccinyldiCQA II	14.48	615	453 (Q), 353, 191
Other hydroxycinnamic acids				
17	Caffeic acid [a]	6.31	179	135 (Q), 134
18	Caffeoyl-hexoside	2.81	341	179 (Q), 135
19	Ferulic acid [a]	9.45	193	134 (Q), 178
20	Isoferulic acid [a]	9.59	193	178 (Q), 134
21	*p*-Coumaric acid [a]	8.70	163	119 (Q), 93
**Flavonoids**				
Apigenin derivatives				
22	Apigenin [a]	17.82	269	151 (Q), 149, 117
23	Apigenin 7–*O*–glucoside [a]	12.56	431	269 (Q), 311, 283
24	Apigenin 7–*O*–glucuronide [a]	12.86	445	269 (Q), 113, 85
25	Apigenin 6,8-di-C-glucoside (Vicenin-2) [a]	8.56	593	353 (Q), 383, 473
Luteolin derivatives				
26	Luteolin [a]	15.83	285	133 (Q), 151
27	Luteolin 7–*O*–glucoside [a]	10.32	447	285 (Q), 327
28	Luteolin 7–*O*–glucuronide [a]	10.41	593	285 (Q), 327
29	Luteolin acetylglucoside	13.38	489	285 (Q), 284
Quercetin derivatives				
30	Quercetin [a]	15.92	301	151 (Q), 179
31	Quercetin 3-glucoside (Isoquercitrin) [a]	10.25	463	300 (Q), 301, 271
Hesperetin derivatives				
32	Hesperetin 7-rutinoside (Hesperidin)[a]	12.80	609	301 (Q), 342

Nº	(Poly)phenolic compound	R_t (min)	$[M–H]^−$ (*m/z*)	Fragment ions (*m/z*)
**Catabolites**				
33	3-Hydroxyphenylacetic acid [a]	6.71	151	107 (Q), 93
34	2,5-Dihydroxybenzoic acid [a]	4.42	153	108 (Q), 109
35	Dihydrocaffeic acid [a]	5.60	181	59 (Q), 137
36	3,4-Dihydroxybenzoic acid (protocatechuic acid) [a]	2.22	153	109 (Q), 91, 81
37	3-(3-Hydroxyphenyl)propionic acid [a]	9.20	165	121 (Q), 119
38	4-Hydroxybenzoic acid [a]	4.12	137	93 (Q)
39	Phenylacetic acid [a]	10.20	135	91 (Q), 89
40	1,2-Dihydroxybenzene [a]	3.13	109	108 (Q), 91
41	3-Hydroxybenzoic acid [a]	6.21	137	93 (Q), 109

R_t, retention time; *m/z*, mass-to-charge ratio; $[M–H]^−$, Negatively charged molecular ion; Q, quantifier transition

[a] Compounds identified and quantified with their own pure reference standard

Figure S1. Cytotoxicity (MTT assay) of raw and *sous-vide* cooked red cardoon after (a) gastrointestinal digestion in differentiated Caco-2 cells, and after (b) 8 h and 24 h of colonic fermentation in HT-29 cells, with and without LPS stimulation. Cells were incubated with supernatants of the digested or fermented cardoon samples or the negative fermentation control (NFC) for 1 h, and then in the absence or presence of LPS (5 μg/mL in differentiated Caco-2 cells; 0.1 μg/mL in HT-29 cells) for further 48 h, without removing the previous treatment. Results are expressed as mean % MTT reduction relative to the control (untreated cells) ± SD (n = 3 experiments).

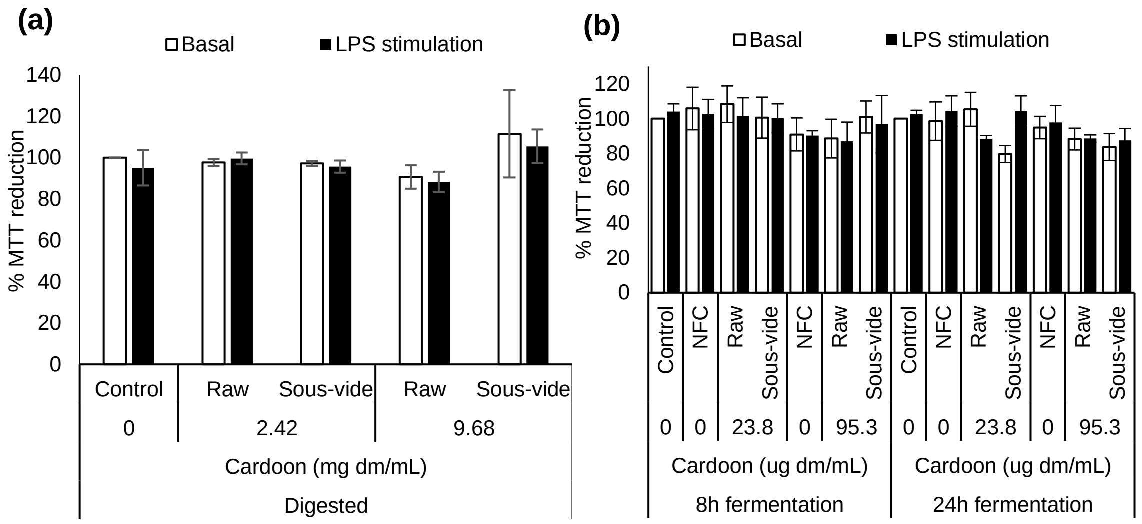

Supplement: Supplementary file 1 — jf1c03014_si_001.pdf [file jf1c03014_si_001.pdf]
